# Barriers and Facilitators to the Implementation of Virtual Reality Interventions for People With Chronic Pain: Scoping Review

**DOI:** 10.2196/53129

**Published:** 2024-05-15

**Authors:** Alexander Elser, Marina Lange, Christian Kopkow, Axel Georg Schäfer

**Affiliations:** 1 Faculty of Social Work and Health HAWK Hildesheim/Holzminden/Göttingen University of Applied Sciences and Arts Hildesheim Germany; 2 Department Therapy Science I Faculty 4 for Human Sciences Brandenburg University of Technology Cottbus – Senftenberg Senftenberg Germany

**Keywords:** virtual reality, VR, chronic pain, implementation science, scoping review, barriers, facilitators

## Abstract

**Background:**

Chronic pain is a growing health problem worldwide with a significant impact on individuals and societies. In regard to treatment, there is a gap between guideline recommendations and common practice in health care, especially concerning cognitive and psychological interventions. Virtual reality (VR) may provide a way to improve this situation. A growing body of evidence indicates that VR therapy has positive effects on pain and physical function. However, there is limited knowledge about barriers and facilitators to the implementation of VR interventions for people with chronic pain in health care settings.

**Objective:**

The aim of this study was to identify and analyze the barriers and facilitators involved in implementing VR interventions for people with chronic pain.

**Methods:**

We conducted a scoping review of the German and English literature using the MEDLINE, Cochrane Central Register of Controlled Trials, CINAHL, PEDro, LILACS, and Web of Science (inception to November 2023) databases, including quantitative, qualitative, and mixed methods studies reporting barriers and facilitators to the implementation of VR interventions for people with chronic pain, as reported by patients or health care professionals. Two reviewers systematically screened the abstracts and full texts of retrieved articles according to the inclusion criteria. All mentioned barriers and facilitators were extracted and categorized according to the Theoretical Domains Framework (TDF).

**Results:**

The database search resulted in 1864 records after removal of duplicates. From the 14 included studies, 30 barriers and 33 facilitators from the patient perspective and 2 facilitators from the health care professional perspective were extracted. Barriers reported by people with chronic pain were most frequently assigned to the TDF domains environmental context (60%) and skills (16.7%). Most facilitators were found in three domains for both the patients and health care professionals: beliefs about consequences (30.3%), emotions (18.2%), and environmental context (18.2%).

**Conclusions:**

The findings of this review can inform the development of strategies for future implementations of VR interventions for people with chronic pain. Additionally, further research should address knowledge gaps about the perspective of health care professionals regarding the implementation of VR interventions for people with chronic pain.

## Introduction

Chronic pain is defined as persistent or recurrent pain lasting longer than 3 months [1]. Chronic pain is an increasingly prevalent health condition worldwide, as three of the primary contributors to years lost to disability in recent decades are chronic pain conditions (back pain, musculoskeletal disorders, and neck pain) [2,3]. Estimated pooled prevalence rates for chronic pain in adults vary across studies from 20.5% in the United States [4] to 28.3% in Germany [5], 34% in the United Kingdom [6], and 48.1% in Chile [7]. High prevalence of chronic pain is not only found in industrial nations but also in low- and middle-income countries, where the prevalence ranges from 13% to 49.4% [8]. Chronic pain affects not only adults but also has a significant prevalence in children, adolescents, and young adults, ranging from 8% to 23% [8-10]. Common consequences of chronic pain include physical disability, psychological distress, and reduced quality of life [3,11]. Furthermore, chronic pain affects relationships and self-esteem and is associated with higher rates of divorce and suicide [12,13]. From a societal perspective, chronic pain places an enormous financial burden on health care systems. In Australia, the financial costs associated with chronic pain were estimated to be ~US $57.1 billion in 2018 [14]. In the United States, the Institute of Medicine estimated that the annual cost of chronic pain, including medical costs and lost productivity, was US $560 billion to US $635 billion in 2010 [15]. In Germany, chronic pain was estimated to cost at least US $63.7 billion annually [16]. At the same time, the care situation for people with chronic pain is characterized by a shortage of health care specialists, resulting in an inadequate supply of treatments [17], particularly of psychotherapy [18]. In contrast, the guidelines for chronic pain explicitly recommend interdisciplinary multimodal pain management, including cognitive and psychological interventions [19].

Virtual reality (VR) is a relatively new nonpharmacological modality to help people suffering from chronic pain, which can also help to improve the care situation [20]. VR treatment for people with chronic pain includes VR games, mindfulness-based interventions, practical exercises, and visual illusions [21]. A meta-analysis showed large effects of VR interventions on pain (standardized mean difference [SMD] 1.6, 95% CI 0.83-2.36) and body functioning (SMD 1.4, 95% CI 0.13-2.67) in people with chronic pain [21]. Although the mechanisms underlying the observed benefits of VR for chronic pain are not yet fully understood, distraction of the patient and embodiment have been discussed as possible explanations for changes in outcomes [20]. Distraction is based on the limited capacity of people to simultaneously attend to different stimuli [22]. It is assumed that attention that would normally be focused on pain is redirected to the VR experience, thereby reducing or eliminating the perception of pain [23]. Embodiment describes the experience of the virtual body in virtual space and can lead to a change in the perception of the physical body and the body matrix, which can have a positive effect on pain perception and physical activity in people with chronic pain [24]. Other mechanisms, including the gamification of exposure to feared movements through the VR [25] and accelerated time perception in VR [26], have also been proposed to have an influence on chronic pain.

VR can therefore be seen as a promising therapeutic option for people with chronic pain. However, there has been no large-scale implementation of this technology in the health care of people with chronic pain. Previous research has shown that organizational structures and the VR technology itself are barriers to the implementation of VR interventions in various health care settings [27-29]. Regarding the use of VR in physiotherapy, due to technical limitations, lack of protocols for VR interventions, and patient-related factors, VR itself seems to be the main barrier [30]. Conversely, staff and health care professionals may act as facilitators, as they reduce the anxiety of new technologies and can change patients’ attitudes toward VR. Health care professionals are also generally interested in using VR in rehabilitation [28-30]. However, people with chronic pain are a group with unique characteristics and diverse impairments, as they may experience pain-related fears and fear of movement, and often have maladaptive coping strategies, mental disorders such as depression or anxiety [31], or cognitive impairments [32]. Since these factors may influence the implementation of VR interventions, it is essential to identify barriers and facilitators for this population in using VR to derive a targeted implementation strategy.

A systematic implementation strategy is necessary to enable large-scale successful implementation and use of VR interventions for people with chronic pain. This requires a comprehensive review of all known barriers and facilitators. The Theoretical Domains Framework (TDF) offers an approach to systematically examine barriers and facilitators toward the development of an implementation strategy [33]. The TDF is an implementation framework for behavioral change that incorporates 128 theoretical concepts derived from 33 different behavior change theories and organizes them into 14 domains into which the barriers and facilitators can be classified [33]. The findings gained in this way can be used to support implementation efforts. For example, this approach was used to support the implementation of stratified care for people with nonspecific low back pain in Canada [34], and was also used to inform the development and implementation of digital tools in a bariatric surgery service [35].

Therefore, the aim of this scoping review was to systematically identify and categorize barriers and facilitators to the implementation of VR interventions for people with chronic pain. The identified barriers and facilitators will provide a basis for recommendations for the successful integration of VR interventions into clinical practice, future development of VR interventions, and future implementation studies in the field of chronic pain management.

## Methods

### Study Design and Registration

A scoping review was conducted to comprehensively search and synthesize the published literature on barriers and facilitators reported by patients and health care professionals in implementing VR interventions for the treatment of people with chronic pain. The methodological background for this scoping review is based on the five steps outlined by Arksey and O’Malley [36] and the methodological guidance for conducting scoping reviews published by the Joanna Briggs Institute [37]. Reporting follows the PRISMA-ScR (Preferred Reporting Items for Systematic Reviews and Meta-Analyses Extension for Scoping Reviews) guidelines; the PRISMA-ScR checklist can be found in Multimedia Appendix 1 [38]. The scoping review was registered with the Open Science Framework [39].

### Search Strategy, Eligibility Criteria, and Selection of Evidence Sources

A database-specific literature search was conducted in the electronic databases MEDLINE (through PubMed), Cochrane Central Register of Controlled Trials, CINAHL, PEDro, LILACS, and Web of Science on November 1, 2022. A search strategy was developed using the keywords “chronic pain,” “virtual reality,” and “implementation.” The detailed search string for each database can be found in Multimedia Appendix 2. Additionally, one reviewer (AE) screened the reference lists of the included studies.

The search results were combined and uploaded to CADIMA, a web application that assists in conducting and documenting the evidence synthesis process [40], which we used for the selection process. After removing duplicates, two authors (AE and ML) independently screened the titles and abstracts of identified publications.

The initial inclusion criteria for publications were: (1) use of quantitative, qualitative, or mixed method study designs; (2) involves people with any type of chronic pain; (3) the treatment was a VR intervention; (4) published in the English or German language; and (5) reported implementation outcomes. The exclusion criterion was studies involving children (aged<18 years).

Two reviewers (AE and ML) tested the inclusion and exclusion criteria by screening the titles and abstracts of a random sample of 25 publications to ensure consistent use. If agreement was below 75%, the criteria were adjusted [41]. After title and abstract screening, the reviewers (AE and ML) discussed refining the criteria for full-text screening. As a result, the criterion to include only studies that specifically reported barriers or facilitators as reported by patients or health care professionals as implementation outcomes was added. Barriers were defined as any factors that inhibit or negatively influence patients’ use of a VR intervention. Facilitators were defined as all factors that enhance or positively influence patients’ use of a VR intervention. Barriers and facilitators had to be self-reported by patients or health care professionals. The two reviewers (AE and ML) independently screened the full texts. Disagreements throughout the review process were resolved by discussion between the two reviewers.

### Data Charting Process

One reviewer (AE) extracted the data into a custom data template created for the purpose of this scoping review (see Multimedia Appendix 3). A second reviewer (ML) reviewed all extracted data and commented on discrepancies, which were resolved through discussion. We extracted study characteristics (title, authors, year of publication, design, population, and sample size), intervention characteristics (setting, type of intervention), and barriers and facilitators (separately for patients and health care professionals). From qualitative studies, all barriers and facilitators reported by patients or health care professionals were extracted. For quantitative studies, barriers and facilitators were extracted if ≥50% of participants agreed that this factor had an influence on the implementation of VR interventions [42].

### Collating, Summarizing, and Reporting

The resulting data were transferred into MAXQDA Plus 2022 (VERBI software, 2021) to code and categorize the barriers and facilitators separately for patients and health care professionals according to the domains of the TDF (see Multimedia Appendix 4). After coding of the barriers and facilitators by two reviewers (AE and ML), inconsistencies were resolved through discussion. Extracted barriers and facilitators could be categorized in more than one domain.

After evaluation of the number of barriers and facilitators assigned to each domain of the TDF, separately for patients and health care professionals, the most common barriers and facilitators were analyzed to determine underlying themes.

## Results

### Study Selection

**Figure 1:**
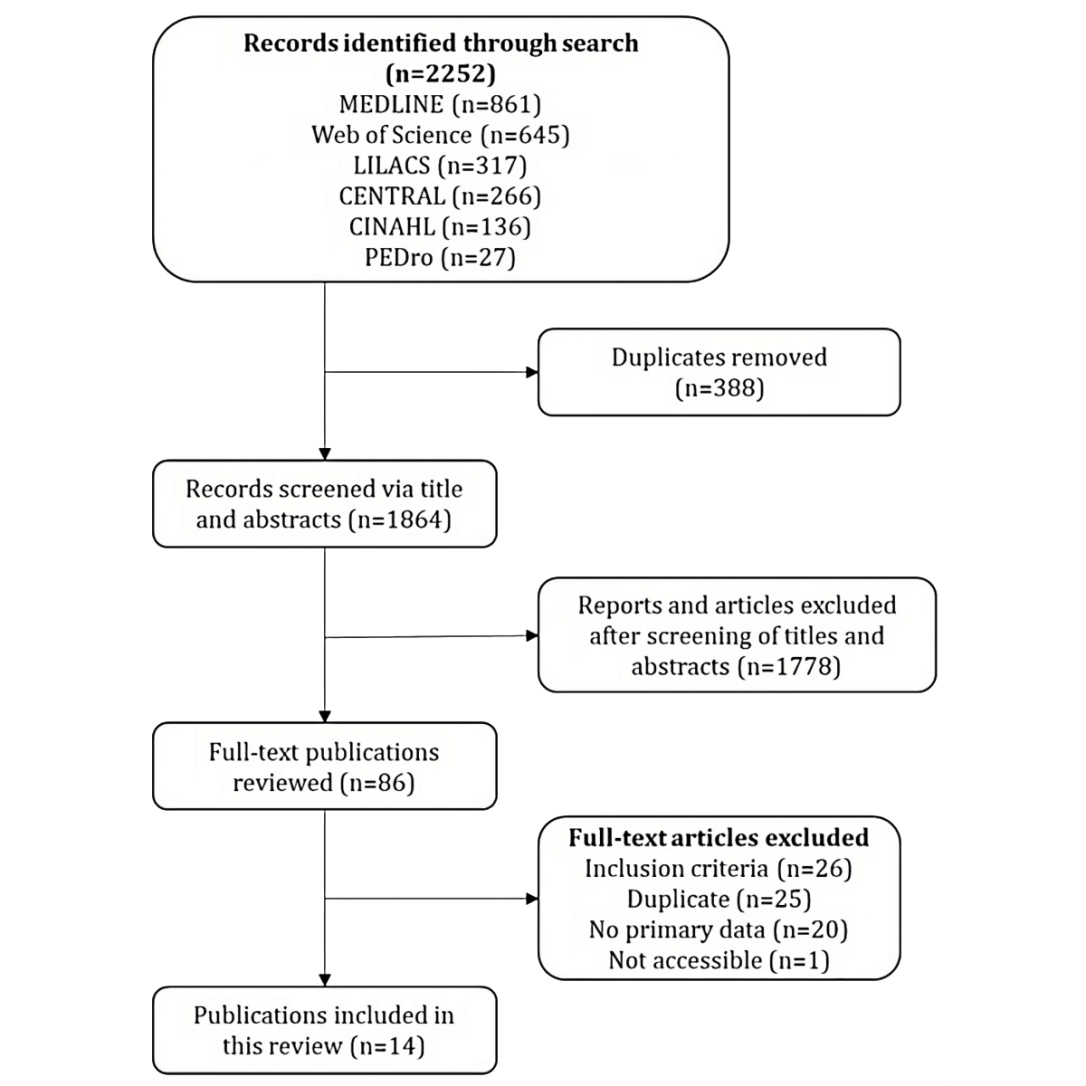
Flowchart of search and screening results.

The database search resulted in 2252 publications. After removal of 388 duplicates, 1864 titles and abstracts were screened. Of those, 86 publications met the inclusion and exclusion criteria and were subject to screening of the full text. Among these 86 publications, 72 were excluded because they did not meet the inclusion criteria, were duplicates, did not provide primary data, or were not accessible. Duplicates occurred again in the screening of full texts because the initial removal of duplicates before the screening of titles and abstracts was based solely on the DOI. However, some publications were not recognized by CADIMA in this step due to missing DOIs. Finally, 14 studies were included in the qualitative analysis. The entire selection process is shown in the PRISMA-ScR flowchart in Figure 1.

### Description of Included Studies

Of the 14 studies, there were 8 mixed methods studies [43-50], four qualitative studies [51-54], and two quantitative studies [55,56]. All studies reported barriers and facilitators from the patient perspective, whereas one study also reported barriers and facilitators from the health care professional perspective [53]. The included studies were published between 2013 and 2022, with 9 studies published in 2020 or later [43-46,51-53,55,56]. The sample size of the studies ranged from 7 [49] to 84 [50] participants, with the mean age ranging from 35.86 [49] to 81.85 [55] years. The studies included various VR interventions such as a 5-minute nature relaxation video [46], physically active tasks [43], and specifically developed interventions with guided exercises for focused attention and open awareness [50]. For more information on the characteristics, study settings, and VR interventions of the included studies, please refer to Multimedia Appendix 5 [43-56].

### Overview of Identified Barriers and Facilitators

A total of 65 barriers and facilitators were identified. Among these, there were 30 (46%) barriers and 33 (51%) facilitators from the patient perspective and 2 (3%) facilitators reported from the health care professional perspective. All identified barriers and facilitators are summarized for each TDF domain in Multimedia Appendix 6.

### Barriers From the Patient Perspective

The 30 barriers identified from the patient perspective were categorized into six different TDF domains (Table 1). The other eight TDF domains did not address the barriers identified from the patient perspective.

**Table 1:** Barriers and facilitators from the patient perspective assigned to Theoretical Domains Framework (TDF) domains.

TDF domains	Barriers (n=30), n (%)	Facilitators (n=33), n (%)
Environmental context and resources	18 (60)	6 (18)
Skills	5 (17)	1 (3)
Memory, attention, and decision processes	2 (7)	0 (0)
Emotion	2 (7)	6 (18)
Beliefs about consequences	1 (3)	10 (30)
Reinforcement	0 (0)	4 (12)
Knowledge	0 (0)	2 (6)
Behavioral regulation	0 (0)	2 (6)
Optimism	0 (0)	1 (3)
Beliefs about capabilities	0 (0)	1 (3)

One of the two most important domains was the *environmental context and resources* (ECR) domain, which included the most barriers from the patient perspective. Three main themes emerged (Table 2). The first was related to the VR devices themselves, with barriers such as the devices being too heavy, too expensive, not detecting all movements, and problems when people with chronic pain were wearing glasses. Furthermore, insufficient support during implementation was perceived as a barrier. The second theme was that the VR software made people with chronic pain feel sick or caused more pain. In addition, the tutorial of the software was considered to be too difficult. Finally, notable events included technical problems, problems with use due to physical impairments, and patients being in too much pain to use.

Within the domain *skills*, we identified two main barrier-related themes: (1) gaming skills, as the software was too difficult to use and patients without previous experience in playing video games had difficulties controlling the game; and (2) other skills, in which the main barrier was language if the patient’s first language was not the same language as that used in the software.

**Table 2:** Main barrier-related themes from the patient perspective according to Theoretical Domains Framework domains.

Themes			Quote/description		References
**Environmental context and resources**					
	VR^a^ devices	“A negative factor was that the VR glasses were heavy to wear”		Glavare et al [45]	
	VR software	“Yeah, that would really have to be under guidance, yeah. [ …] So they [peers] wouldn’t be able to do it alone”		Stamm et al [52]	
	Notable events	“I’ve got really bad arthritis too at the moment so holding onto those [hand controls] was an issue”		Kelly et al [51]	
**Skills**					
	Gaming skills	“I had some trouble figuring out which controls to use to move around so um I’ve never played computer games before and maybe that had something to do with it. I felt like a total idiot totally frustrated and not able to catch onto what to do”		Garrett et al [48]	
	Other skills	The exceptions were those whose first language was not English and who described difficulties in understanding game instructions		Tuck et al [43]	

^a^VR: virtual reality.

### Facilitators From the Patient Perspective

The 33 facilitators were assigned to nine different domains of the TDF (Table 1). No facilitators were assigned to the other five domains of the TDF.

The most frequently identified facilitators were categorized in the domain *beliefs about consequences*. A closer look at this domain revealed the following three main themes: (1) positive expectations in regard to therapy effects, (2) the VR interventions are helpful for rehabilitation, and (3) the VR interventions support doing therapy regularly (Table 3). Among the positive expectations for treatment effects, pain, feelings of anxiety and depression, as well as expectations that VR interventions are superior to conventional therapy were mentioned as facilitators. VR interventions were reported to be helpful for rehabilitation because they improved the mood, well-being, and concentration of people with chronic pain. Similarly, people with chronic pain stated that using VR interventions would increase their adherence with the health behavior and that they would use it on a regular basis.

**Table 3:** Main facilitator-related themes from the patient perspective according to Theoretical Domain Framework domains.

Themes			Quote/description		Reference
**Beliefs about consequences**					
	Positive expectations	“I had really high hopes...I thought it might actually take my pain away”		Tuck et al [43]	
	Helpful for rehabilitation	“I’ve taken opiates for 40 years and they don’t work as well as what the virtual reality did”		Kelly et al [51]	
	Increase adherence	“Especially in the future, you could have thousands of different situations that you could immerse yourself in, for as much time as you want in the day”		Garrett et al [53]	
**Environmental context and resources**					
	VR^a^ devices	Several participants noted that flexibility in the position of use and brevity of time in the experience had helped manage or entirely avoid such discomfort: “I’m not in as much pain when I’m seated as when I’m standing, so it was quite easy for me to do the movements”		Kelly et al [51]	
	Supervising therapist	“I think it makes you feel better that it’s a trained physiotherapist. You knew they had that background and it just fills you with confidence a bit more”		Tuck et al [43]	
	Gamification	“Positive factors were that VR added a dimension of playfulness and gaming to the exercise”		Galavare et al [45]	
**Emotion**					
	Fun and enjoyment	The competition against the computer opponents increased engagement and several participants mentioned the feeling of satisfaction they got when they performed well		Mortensen et al [54]	
	Novel and unknown experiences	“you’re enjoying yourself, you can do things you’ve never experienced before, obviously you’re going to do it”		Kelly et al [51]	

^a^VR: virtual reality.

One of the second most frequently identified facilitators was ECR, with three main themes: (1) VR devices, (2) a supervising therapist, and (3) gamification. The VR devices serve as a facilitator because they are simple to use for people with chronic pain, easily adjustable, and can be used in different positions. Similarly, a supervising therapist is considered to facilitate the implementation of a VR intervention. The gamification of therapy through VR interventions was also perceived as a facilitating factor by people with chronic pain.

The second most frequently identified facilitator was classified under the *emotion* domain of the TDF. Within this domain, two main themes were derived: (1) fun and enjoyment, in which people with chronic pain reported that VR interventions triggered positive emotions and evoked a high level of satisfaction; and (2) novel and unknown experiences that the people with chronic pain are not able to experience in the real world.

### Facilitators From the Health Care Professional Perspective

Only two facilitators from the health care professional perspective were identified, which were assigned to the domains *ECR* and *beliefs about consequences*. Health care professionals indicated that the opportunity to be with the patient during the VR intervention and to be able to intervene in adverse events supports its implementation. Another facilitating factor from the health care professional perspective was that the VR intervention allows patients to practice everyday situations in therapy, such as working in the garden.

## Discussion

### Overview

The aim of this scoping review was to identify and categorize barriers and facilitators associated with the implementation of VR interventions for people with chronic pain, using the TDF. From the 14 included studies [43-56], a total of 65 barriers and facilitators from the patient perspective and two facilitators from the health care professional perspective were identified. The main barriers from the patient perspective to use VR interventions for chronic pain were assigned to the domains *environmental context* and *resources and skills*. However, the domains *ECR*, *beliefs about consequences*, and *emotions* also included facilitators that increased the use of VR interventions from a patient perspective. Health care professional perspectives are poorly researched, with only one study [52] found on this topic. To our knowledge, this is the first scoping review summarizing barriers and facilitators to the implementation of VR interventions for people with chronic pain.

### Selection of a VR Device

At first glance, a contradictory result of this study is that the *ECR* domain includes barriers as well as facilitators to the implementation of VR interventions for chronic pain. However, since VR devices and VR software emerged as major themes within the barriers and facilitators in this domain, an important step in implementing VR interventions appears to be the selection of an appropriate VR device and VR software for patients with chronic pain and in consideration of their actual conditions. This decision may be particularly important for people with chronic neck pain, as they may be more sensitive to the weight of VR devices, which could lead to an increase in pain [49]. Although future technological developments of VR devices with lower weight might improve this limitation, the use of VR devices for people with chronic neck pain will remain an individual decision depending on individual tolerance. If these steps are taken carefully, it is possible that the chosen VR device and the VR intervention itself will act as a facilitator in the implementation process. These findings support three proposed aspects to be considered when preparing a VR therapy: the right VR intervention at the right time and with the right patient [57]. These findings are consistent with published recommendations to adopt a participatory approach involving the patients themselves throughout the development process of VR interventions to consider all of the above aspects at an early stage [58].

### VR Skills

A second important TDF domain including barriers was *skills*, relating specifically to the patient’s gaming skills and language skills. Both can be addressed in software development, such as by participatory developed tutorials or using plain language. These recommendations are partly reflected in the recommendations for the participatory development of VR interventions [58] and are also in line with a previous review, which argued for providing sufficient time to learn and use the new technology for patients and health care professionals [27]. However, our findings emphasize the importance of developing and providing plain-language options in VR interventions for people with chronic pain, potentially due to their shorter attention spans and greater susceptibility to interruption [59], as well as other mental health concerns such as psychological distress [11], anxiety, and depression [31]. For existing interventions, these barriers can be addressed with an implementation strategy. As part of such a strategy, special attention should be given to competencies of health care professionals related to the use of VR to enable them to teach the acquired skills to their patients with individual needs [29]. Additionally, for a successful implementation, it is important that health care professionals are positive about the digital technology [60] and perceive it as user-friendly [61]. Thus, a key aspect of implementing VR in the treatment of chronic pain is adequate training of the health care professionals who will provide the VR interventions to people with chronic pain.

No barriers in regard to game design quality, such as poor graphics or boring games, were reported by people with chronic pain or health care professionals, which was a somewhat surprising finding. Considering the publication dates of the literature retrieved and our own experience with VR interventions, it would have been conceivable that the grade of immersion or perceived difference between the virtual world and the real world could still be experienced as a barrier to using VR interventions.

### VR Treatment Expectations

Existing positive expectations regarding pain improvement and rehabilitation facilitate the implementation of VR interventions for people with chronic pain [43,51,53,55,56]. A positive belief in VR interventions seems to result in more satisfaction with the outcome of therapy in general [30] and has an impact on cooperation and outcomes in people with chronic pain in general [62]. When implementing VR interventions for people with chronic pain, this positive belief can be used and facilitated by educating patients about the positive effects of the intervention and presenting best-practice examples.

Another theme that emerged within the domain beliefs about consequences is that VR interventions could increase treatment adherence because VR helps people with chronic pain to improve health behaviors and their ability to focus on tasks [56]. In addition, patients see the possibility that in the future they will be able to choose from many different virtual scenarios in which they can immerse themselves to help with their pain [53].

Lack of patient adherence is a common problem associated with poorer treatment outcomes [63,64] and VR may be a viable option to reduce this problem. Our results are in line with a previous review, which showed that VR can encourage patients to adhere to treatment [30]. VR and its potential impact on adherence may facilitate high-intensity therapy and thereby improve outcomes, as a network meta-analysis showed that high-intensity therapy in particular can have a positive impact on outcomes in chronic pain therapy [65].

The themes fun and enjoyment and having novel experiences provide an explanation for the above-mentioned good adherence to VR interventions. The ability of VR to provide novel experiences for people with chronic pain has also been highlighted in other studies [30]. Furthermore, positive emotions such as fun and enjoyment may themselves have a positive impact, considering that negative emotions are a risk factor for the development and maintenance of chronic pain [66].

### Perspective of the Health Care Professionals

In our scoping review, we were only able to identify one study that focused on facilitators from the perspective of health care professionals, who naturally play a crucial role in the implementation of digital interventions [60]. Health care professionals mentioned that VR is a good opportunity to treat people with chronic pain in everyday situations and that they want to be close to the patients during the treatment [53].

### Integration With Existing Literature

When comparing the findings of this review with findings from other reviews looking at the implementation of VR interventions in various health care settings [29], rehabilitation [27], and physical therapy [30], it is notable that the identified themes differ only in terms of the details and cover mostly similar topics such as the barrier of appropriate VR interventions and VR devices for the individual patient, as well as the facilitators of having a strong belief in the efficacy of these interventions. This preliminary finding suggests that implementing VR interventions for people with chronic pain is not fundamentally different from implementation in other settings; however, due to the nature of chronic pain, cognitive and functional impairments should be taken into account. Additionally, since the health care professional perspective is not fully understood, further research on this aspect is necessary.

### Limitations

One limitation of this scoping review is that classification of barriers and facilitators into TDF domains may be subjective, although we aimed to minimize subjectivity by standardized procedures using a coding guideline based on TDF domains with two independent reviewers. In this scoping review, barriers and facilitators were ranked according to how often they were mentioned in the included studies. However, this may not necessarily reflect their importance. Barriers and facilitators mentioned only once may nevertheless be the most important factor in a particular implementation setting. In particular, barriers and facilitators in quantitative studies were included if they had more than 50% agreement in the study, whereas all barriers and facilitators mentioned in qualitative studies were included. This may impact the distribution of barriers and facilitators.

The review process did not include a critical appraisal of the studies; although this is not standard practice for scoping reviews, it might be considered a limitation regarding the quality of the evidence. Furthermore, only studies published in German or English were included. This may limit conclusions about implementation in other countries.

### Recommendations

Our findings provide a comprehensive overview of the barriers and facilitators to implementing VR interventions for people with chronic pain in the existing literature. Based on the identified barriers, the development of VR devices and VR interventions should address the perspectives of both people with chronic pain and health care professionals. This could reduce language, cognitive, or physical barriers that are important for patients with specific impairments.

Based on the identified barriers and facilitators, systematic and targeted implementation strategies for VR interventions for people with chronic pain can be developed. For example, the barrier of lack of skills can be reduced by offering targeted training to health care professionals, and positive expectations of VR interventions can be reinforced, such as by displaying a poster about the positive effects of VR in the waiting room. In addition, future research on VR interventions and implementation should pay more attention to the perspective of health care professionals to gain better insight into the values and needs of these critical stakeholders. This can be achieved through an implementation study that includes a formative evaluation of the implementation steps with a focus on health care professionals and their experiences in the process.

## Appendix

Multimedia Appendix 1 PRISMA-ScR (Preferred Reporting Items for Systematic Reviews and Meta-Analyses extension for Scoping Reviews) checklist.

Multimedia Appendix 2 Search strategy.

Multimedia Appendix 3 Data extraction sheet.

Multimedia Appendix 4 Description of domains in the Theoretical Domains Framework.

Multimedia Appendix 5 Description of the included studies.

Multimedia Appendix 6 Summary of all identified barriers and facilitators according to Thematic Domains Framework domains.

## Abbreviations

ECR: environmental context and research
PRISMA-ScR: Preferred Reporting Items of Systematic Reviews and Meta-Analyses extension for Scoping Reviews.
SMD: standardized mean difference
TDF: Theoretical Domains Framework
VR: virtual reality

## Data Availability

The data sets generated and analyzed during this study are available from the corresponding author on reasonable request.
