# Supplementary material for: Barriers and Facilitators to the Implementation of Virtual Reality Interventions for People With Chronic Pain: Scoping Review
Source: JMIR XR Spat Comput. 2024 May 15;1:e53129. doi: 10.2196/53129 (PMC12671291; doi:10.2196/53129)
Supplement: Multimedia Appendix 2 [file xr_v1i1e53129_app2.docx]

**Multimedia Appendix 2. Search strategy.**

**PubMed**

| 1 | ((((((((((chronic pain*)) OR (widespread pain)) OR (chronischer Schmerz)) OR (chronische schmerzen)) OR ("Chronic Pain"[Mesh])) OR (chronic pain patient*)) OR (persistent pain)) OR (intractable pain)) OR ("Pain, Intractable"[Mesh])) OR (chronic pain management) |
| --- | --- |
| 2 | (((((((((((((virtual real*) OR (virtual technolog*) OR (virtual game*) OR (virtual environment)) OR ("Virtual Reality"[Mesh])) OR ("Virtual Reality Exposure Therapy"[Mesh])) OR (virtual reality exposure therapy)) OR (VR)) OR (augmented reality)) OR (computer simulation)) OR (immersi*)) OR (head mounted display)) OR (simulated reality)) OR (simulated environment)) OR (virtual simulation)) OR (simulation technology) |
| 3 | ((((((((((implement*) OR (barrier* OR facilitat* OR support* or challeng*)) OR (Feasibility)) OR (accept*)) OR (compatib*)) OR (usefulness)) OR (understanding)) OR (motivators)) OR (experienc*)) OR (perspective*)) |
| 4 | 1 AND 2 AND 3 |

**Cochrane Central Register of Controlled Trials**

ID Search

#1 MeSH descriptor: [Chronic Pain] explode all trees

#2 chronic pain*

#3 widespread pain*

#4 chronische* Schmerz*

#5 chronic pain patient*

#6 persistent pain

#7 intractable pain

#8 chronic pain management

#9 #1 OR #2 OR #3 OR #4 OR #5 OR #6 OR #7 OR #8

#10 MeSH descriptor: [Virtual Reality] explode all trees

#11 virtual real*

#12 virtual technolog*

#13 virtual game*

#14 virtual environment

#15 virtual reality exposure therapy

#16 MeSH descriptor: [Virtual Reality Exposure Therapy] explode all trees

#17 augmented reality

#18 computer simulation

#19 immersi*

#20 head mounted display

#21 simulated reality

#22 simulated environment

#23 virtual simulation

#24 simulation technology

#25 #10 OR #11 OR #12 OR #13 OR #14 OR #15 OR #16 OR #17 OR #18 Or #19 OR #20 OR #21 OR #22 OR #23 OR #24

#26 MeSH descriptor: [Implementation Science] explode all trees

#27 implement*

#28 barrier*

#29 facilitat*

#30 support*

#31 challeng*

#32 feasibility

#33 acceptability

#34 compatib*

#35 usefulness

#36 understanding

#37 motivators

#38 experienc*

#39 perspective

#40 #26 OR #27 OR #28 OR #29 OR #30 OR #31 OR #32 OR #33 OR #34 OR #35 Or #36 OR #37 Or #38 OR #39

#41 #9 AND #25 AND #40

**CINAHL**

#1 (mh:(chronic pain)) OR (chronic pain) OR (widespread pain) OR (chronischer schmerz) OR (chronische Schmerzen) OR (chronic pain patient*) OR (persitent pain) OR (intractable pain) OR (mh:(Pain, Intractable)) OR (chronic pain management)

#2 (mh:(Virtual reality)) OR (virtual real*) OR (virtual technolog*) OR (virtual game*) OR (virtual environment) OR (virtual reality exposure therapy) OR (mh:(virtual reality exposure therapy)) OR (augmented reality) OR (computer sumlation) OR (immseriv*) OR (head mounted display) OR (simulated reality) OR (simulated environment) OR (simulated technology)

#3 (mh:(Implementation Science)) OR (implement*) OR (barrier*) OR (facilitat*) OR (support*) OR (challeng*) OR (feasibility) OR (accept*) OR (compatib*) OR (usefulness) OR (understanding) OR (motivators) OR (experienc*) OR (perspective*)

#1 AND #2 AND #3

**PEDro**

Chronic pain AND virtual reality

**LILACS**

#1 (mh:(chronic pain)) OR (chronic pain) OR (widespread pain) OR (chronischer schmerz) OR (chronische Schmerzen) OR (chronic pain patient*) OR (persitent pain) OR (intractable pain) OR (mh:(Pain, Intractable)) OR (chronic pain management)

#2 (mh:(Virtual reality)) OR (virtual real*) OR (virtual technolog*) OR (virtual game*) OR (virtual environment) OR (virtual reality exposure therapy) OR (mh:(virtual reality exposure therapy)) OR (augmented reality) OR (computer sumlation) OR (immseriv*) OR (head mounted display) OR (simulated reality) OR (simulated environment) OR (simulated technology)

#3 (mh:(Implementation Science)) OR (implement*) OR (barrier*) OR (facilitat*) OR (support*) OR (challeng*) OR (feasibility) OR (accept*) OR (compatib*) OR (usefulness) OR (understanding) OR (motivators) OR (experienc*) OR (perspective*)

#1 AND #2 AND #3

**Web of Science**

#1 **(((((((ALL=(chronic pain)) OR ALL=(widespread pain)) OR ALL=(chronic pains)) OR ALL=(chronische schmerz*)) OR ALL=(chronic pain patient)) OR ALL=(persistent pain)) OR ALL=(intractable pain)) OR ALL=(chronic pain management)**

**#2 (((((((((((((ALL=(virtual real*)) OR ALL=(virtual technolog*)) OR ALL=(virtual game*)) OR ALL=(virtual environment)) OR ALL=(virtual reality exposure therapy)) OR ALL=(VR)) OR ALL=(augmented reality)) OR ALL=(computer simulation)) OR ALL=(imersi*)) OR ALL=(head mounted display)) OR ALL=(simulated reality)) OR ALL=(simulated environment)) OR ALL=(virtual simulation)) OR ALL=(simulation technology)**

**#3 ((((((((((((ALL=(implement*)) OR ALL=(barrier)) OR ALL=(facilitat*)) OR ALL=(support*)) OR ALL=(challeng*)) OR ALL=(feasibility)) OR ALL=(acceptability)) OR ALL=(compatib*)) OR ALL=(usefulness)) OR ALL=(understanding)) OR ALL=(motivators)) OR ALL=(experienc*)) OR ALL=(perspective*)**

**#1 AND #2 AND #3**
