# Supplementary material for: Barriers and Facilitators to the Implementation of Virtual Reality Interventions for People With Chronic Pain: Scoping Review
Source: JMIR XR Spat Comput. 2024 May 15;1:e53129. doi: 10.2196/53129 (PMC12671291; doi:10.2196/53129)
Supplement: Multimedia Appendix 4 [file xr_v1i1e53129_app4.pdf]

| Domain (definition) | Constructs |
| --- | --- |
| 1. Knowledge | Knowledge (including knowledge of condition/scientific rationale) |
| (An awareness of the existence of something) | Procedural knowledge |
| | Knowledge of task environment |
| 2. Skills | Skills |
| | Skills development |
| | Competence |
| (An ability or proficiency acquired through practice) | Ability |
| | Interpersonal skills |
| | Practice |
| | Skill assessment |
| 3. Social/Professional Role and Identity | Professional identity |
| | Professional role |
| | Social identity |
| | Identity |
| (A coherent set of behaviours and displayed personal qualities of an individual in a social or work setting) | Professional boundaries |
| | Professional confidence |
| | Group identity |
| | Leadership |
| | Organisational commitment |
| 4. Beliefs about Capabilities | Self-confidence |
| | Perceived competence |
| | Self-efficacy |
| (Acceptance of the truth, reality, or validity about an ability, talent, or facility that a person can put to constructive use) | Perceived behavioural control |
| | Beliefs |
| | Self-esteem |
| | Empowerment |
| | Professional confidence |
| 5. Optimism | Optimism |
| | Pessimism |
| (The confidence that things will happen for the best or that desired goals will be attained) | Unrealistic optimism |
| | Identity |

| | |
|---|---|
| 6. Beliefs about Consequences<br><br>(Acceptance of the truth, reality, or validity about outcomes of a behaviour in a given situation) | Beliefs<br>Outcome expectancies<br>Characteristics of outcome expectancies<br>Anticipated regret<br>Consequents |
| 7. Reinforcement<br><br>(Increasing the probability of a response by arranging a dependent relationship, or contingency, between the response and a given stimulus) | Rewards (proximal / distal, valued / not valued, probable / improbable)<br>Incentives<br>Punishment<br>Consequents<br>Reinforcement<br>Contingencies<br>Sanctions |
| 8. Intentions<br>(A conscious decision to perform a behaviour or a resolve to act in a certain way) | Stability of intentions<br>Stages of change model<br>Transtheoretical model and stages of change |
| 9. Goals<br><br>(Mental representations of outcomes or end states that an individual wants to achieve) | Goals (distal / proximal)<br>Goal priority<br>Goal / target setting<br>Goals (autonomous / controlled)<br>Action planning<br>Implementation intention |
| 10. Memory, Attention and Decision Processes<br><br>(The ability to retain information, focus selectively on aspects of the environment and choose between two or more alternatives) | Memory<br>Attention<br>Attention control<br>Decision making<br>Cognitive overload / tiredness |
| 11. Environmental Context and Resources<br><br>(Any circumstance of a person's situation or environment that discourages or encourages the development of skills and abilities, independence, social competence, and adaptive behaviour) | Environmental stressors<br>Resources / material resources<br>Organisational culture<br>Salient events / critical incidents<br>Person x environment interaction<br>Barriers and facilitators |
| 12. Social influences | Social pressure |

| | |
|---|---|
| (Those interpersonal processes that can cause individuals to change their thoughts, feelings, or behaviours) | Social norms<br>Group conformity<br>Social comparisons<br>Group norms<br>Social support<br>Power<br>Intergroup conflict<br>Alienation<br>Group identity<br>Modelling |
| 13. Emotion<br><br>(A complex reaction pattern, involving experiential, behavioural, and physiological elements, by which the individual attempts to deal with a personally significant matter or event) | Fear<br>Anxiety<br>Affect<br>Stress<br>Depression<br>Positive / negative affect<br>Burn-out |
| 14. Behavioural Regulation<br>(Anything aimed at managing or changing objectively observed or measured actions) | Self-monitoring<br>Breaking habit<br>Action planning |
