# Supplementary material for: Barriers and Facilitators to the Implementation of Virtual Reality Interventions for People With Chronic Pain: Scoping Review
Source: JMIR XR Spat Comput. 2024 May 15;1:e53129. doi: 10.2196/53129 (PMC12671291; doi:10.2196/53129)
Supplement: Multimedia Appendix 5 [file xr_v1i1e53129_app5.docx]

Multimedia Appendix 5. Description of the included studies.

| **Authors** | **Year** | **Title** | **Type of study** | **Study sample (patients)** | **Population** | **Setting** | **Intervention** |
| --- | --- | --- | --- | --- | --- | --- | --- |
|  |  |  |  |  |  |  |  |
| Beltran-Alacreu et al [55] | 2022 | A Serious Game for Performing Task-Oriented Cervical Exercises Among Older Adult Patients With Chronic Neck Pain: Development, Suitability, and Crossover Pilot Study | Quantitative | 14 | Age: Mean 81.85 (SD 6.82), Range: 71 - 92  Mini–Mental State Examination score 31.08 (SD 3.01) Neck Disability Index scored 15.77 (SD 8.19)  Chronic neck pain | Residencia Municipal de Griñón  A nursing home in Madrid, Spain | “The main functionality of the software solution was to control a virtual airplane to reach targets using head motions.” |
| Brown et al [46] | 2020 | The use of virtual reality for Peri-procedural pain and anxiety at an outpatient spine clinic injection visit: an exploratory controlled randomized trial | Mixed Methods | 45 | Age: Mean 61.9 (SD 17.7)  Average 7-day pain: 6.0 ± 1.8  Chronicity: 3-6 months: 3 6-12 months: 6 1-5 years: 19 >5 years: 17  Chronic LBP | Medical practice of the principle investigator | “A five-minute nature relaxation video by ECOVR (a non-profit initiative that develops immersive nature content) in VR immersive format in the Oculus Go headset.” |
| Garrett et al [53] | 2020 | Patients perceptions of virtual reality therapy in the management of chronic cancer pain | Qualitative | 12 | Age: Median (Range)  Focus Group 1 59 (37-62) Focus Group 2 58 (45-73)  Average pain score in VAS: Median (Range) Focus Group 1:  4 (3-7) Focus Group 2: 6 (2.5-8; )  6 neuropathic pain 2 fibromyalgia 2 post operation pain 2 undefined | The participants experienced the VR for 45 min in their home through an HTC Vive stereoscopic headset. | “Two of the VR interventions that the participants were exposed to used contemplative mindfulness-based environments, whilst the other two used cognitive problem-solving environments.” |
| Garrett et al [48] | 2017 | Virtual Reality as an Adjunct Home Therapy in Chronic Pain Management: An Exploratory Study | Mixed Methods | 8 | Age: Mean 51 | Patients were recruited by Web-based invitations.  British Columbia, USA  A home-based VR intervention was selected for the study. | “Four categories of VR experience were devised, and VR applications were purposefully selected and tested in advance for potential efficacy by the researchers for use in each of the 4 weeks of the study. Week 1, the participants undertook passive VR experiences where they simply travelled through a VR environment. These included a virtual Iceland, and a boat ride through an artistic experience (Senza Peso). Week 2 were mindfulness and meditative introversion focused VR applications were used, as these have been associated with pain control in other studies. These experiences involved flying through 3D mandalas, or experiences that altered the user’s environment depending where they looked (Sightline). In week 3, active exploratory VR environments were used, where the participant could explore a new environment at will (an underwater environment, the solar system, and a natural environment). In week 4, active problem-solving experiences were used (eg, game type environments requiring participants to solve 3D puzzles).” |
| Glavare et al [45] | 2021 | Virtual Reality Exercises in an Interdisciplinary Rehabilitation Program for Persons with Chronic Neck Pain: a Feasibility Study | Mixed Methods | 12 | Age: Mean 42  Median time with pain: 4.5 years   Chronic neck pain | Interdisciplinary rehabilitation program  The patients were referred to the IRP by their primary care physician. | “After assessment, the patients took part in the IRP. The IRP included education about pain physiology and mechanisms related to chronic neck pain, coping with pain, self-management, the relationship between pain, bodily signs/bodily signals, behavior and emotions, how to balance between activity and rest, relaxation, recovery, body awareness, physical activity, ergonomics, and information on social rights. Each group comprised 8 patients. Each day of the IRP included physical activity, such as training in the gym, cardio training, stability training, walking, Nordic walking, and pool training. The VR exercises were visualized via a mobile phone placed in VR glasses, with a total weight of 0.5 kg. The exercises were guided by an experienced physiotherapist and took approximately 10 min to perform, the whole session lasted approximately 20 min. The VR intervention comprised 3 exercises: 1 Full range of motion of the neck in rotations, flexion and extension by moving a disc via movements of the head and eyes in different predetermined directions. There was no preprogrammed time, and the participant chose the pace. 2 Tracking the disc moving in a predetermined path; a horizontal figure-of-8, without moving outside the path. The preprogrammed time for this exercise was approximately 40 s. 3 Starting with the head in a neutral position, finding the disc, when it appears in different places on the screen, by glancing with the eyes and moving it back to a centred position using their eye gaze , until the head is in the neutral position again. The pre-programmed time for this exercise was approximately 20 s/task.” |
| Kelly et al [51] | 2022 | It made you feel like you've still got it: experiences of people with chronic low back pain undertaking a single session of body image training in virtual reality | Qualitative | 17 | Age: Mean 52 (SD 14)  Average pain duration: 17,5 Years (Range 2-50 Years)  Average pain intensity in a week: 6,2  Roland Morris Disability Index: Mean 57,1 %, Range (16,7-95,8) | Facility for tertiary prevention of chronic pain | “VR-BiT involves the use of VR to facilitate the embodiment of hyper-muscular and hyper-capable avatars. The intervention attempts to facilitate re-encoding of the body as healthy and resilient, while targeting negative self perceptions, and ultimately aiming to reduce pain; Participants in the experimental group were fitted with a VR head mounted display (Oculus Rift S) with connected touch controllers (Oculus, Facebook Technologies, LCC, Menlo Park, USA), and guided by a physiotherapist researcher in interacting with three preexisting VR applications. A boxing application (Creed: Rise to Glory) required participants to make muscle poses, throw air punches, and punch a virtual punching bag. In Avengers Powers Unite, participants became the Incredible Hulk and were instructed to throw air punches and adopt various muscle poses while fixing their attention on their newly adopted musculature. In The Climb, participants were instructed to notice their strength and effortlessness while scaling a virtual cliff. These applications could be completed in a sitting or standing position to suit the tolerance of each participant and were undertaken for approximately six minutes each (total exposure 18 minutes).” |
| Liu et al [56] | 2021 | Preliminary Study of Virtual-reality-guided Meditation for Veterans with Stress and Chronic Pain | Quantitative | 31 | Age: Mean 55,2  Range 20 - 81 years  The majority of participants (87.1%) reported that they were currently experiencing migraines (35.5%), headaches (41.9%), or some other form of chronic pain (80.6%)  A majority of participants (83.8%) reported current concerns related to mental health, such as PTSD (67.7%), general stress (74.2%), acute or chronic anxiety (51.6%), or depression (58.1%). Participants reported a combination of conditions. | Department of Veterans Affairs Polytrauma Headache Center of Excellence  Palo Alto, USA | “All VR meditation sessions were conducted using a portable and Wi-Fi-enabled Oculus Go headset and controller (Facebook Technologies, Irvine, CA, USA).  In the application, the meditation modules were 10-minutes long and used the same preselected ambient music and guided meditation script for all participants, which was centered around the Zen form of meditation. The meditation script instructed participants on an effortless-breathing exercise and guided them in establishing a state of nonjudgmental awareness of their thoughts.” |
| Mortensen et al [54] | 2015 | Women with fibromyalgia's experience with three motion-controlled video game consoles and indicators of symptom severity and performance of activities of daily living | Qualitative | 15 | Age: Mean 49,29 | Aalborg University Campus Esbjerg  Denmark | “Wii and PS3 Move games are played via one or two motion sensitive handheld controllers, depending on the game; whereas Xbox Kinect uses range camera technology to track body movement, and thereby does not involve a controller.” |
| Sarkar et al [44] | 2022 | The feasibility and effectiveness of virtual reality meditation on reducing chronic pain for older adults with knee osteoarthritis | Mixed Methods | 19 | Age: Mean 67.9 (SD 4.6)  number of years of chronic pain: Mean: 18.4 years (SD = 10.9), Range: 3 - 35 years   People with chronic knee osteoarthritis | Brigham and Women's Hospital  Boston, USA | “The subject then participated in a 10-min VR meditation session and was able to customize the experience through various options in the Guided Meditation VR program. The following choices to customize the experience: (1) whether participants wanted to simulate a slow, smooth floating motion through their scene or if they wanted to meditate in a static scene, (2) what type of nature scene they wanted, such as the beach, the forest, outer space, etc., (3) what type of meditation mood they wanted such as energizing, calming, focusing (each was slightly different in the phrases used during the meditation and in the gender of the vocals used to guide the meditation), (4) whether they wanted music in the background. The type of meditation, movement meditation, remained constant for all participants. The movement meditation guided participants through mindful breathing paired with arm movements and focused body awareness.” |
| Stamm et al [52] | 2020 | Virtual reality in pain therapy: a requirements analysis for older adults with chronic back pain | Qualitative | 10 CBP Patients   3 Physical therapists  2 Psychotherapists | Patients: Age: Mean 75.9 years (SD 6.9) old.  Chronic back pain  Von Korff: Grade I: 7 Grade II: 1 Grade III: 2 Grade IV: 0  Short Form (12) Health Survey:  Mean mental health score: 53 (SD 8.0)  Physical health score: 37 (SD 10.7)  Physiotherapists:  2 are employed in a geriatric rehabilitation clinic and 1 in a physiotherapy center.  Experience: 4 - 10 years  Psychotherapists: 1 works in a clinic with focus on acute geriatric medicine and 1 owns a psychotherapy practice Experience: 6.5 - 14 years. | X | “Two applications on the Dell Visor VR118 Headset.”  “In order to create a common understanding for VR in the sample of seniors an exergame user experience prototype was used. In the prototype, the player stands on a platform on a lake and has to collect pink coins. These appear for a short time around the player. The player’s straight posture is determined by the headset height at the beginning of the application, and subsequent malposition of the back turns the player’s surroundings from a colorful landscape to black and white one.”  “The second application was the HoloTour by Microsoft Corporation. The aim was that the player experiences the feeling of immersion in a realistic-looking virtual space.” |
| Tong et al [49] | 2018 | A Case Study: Chronic Pain Patients’ Preferences for Virtual Reality Games for Pain Distraction | Mixed Methods | 7 | Age: Mean = 35.86 (SD = 12.64)  Chronic pain duration: Mean 7.64 years (SD = 7.16) | X | “In the VR condition, HTC Vive HMD and Alienware desktop were used.  As for the four games that were chosen, Call of the Starseed is a calm and slow First-Person VR adventure game in which players need to a few solve puzzles to progress in the narrative. The Witness is a PC title, which is a similar title to Call of the Starseed regarding the intended experience: players solve puzzles in an island. The puzzles in Call of the Starseed requires bodily movements such as bending, which might not be easy for CP patients. Therefore, we also included the game Carpe Lucem, which involves solving physics puzzles in a sitting or standing position. Obduction is an exploration game which has both VR and PC versions. The virtual environment in Obduction has many sci-fi elements.” |
| Tuck et al [43] | 2022 | Active Virtual Reality for Chronic Primary Pain: Mixed Methods Randomized Pilot Study | Mixed Methods | 20 | Age: Mean 40.1 (SD 16.2)  Pain duration: 12-24 months (3), 2-5 years (5), >5 years (12)  Brief pain inventory intensity: mean 8,3 (SD 1,5) Brief pain inventory interference: mean 7,3 (SD 1,6)  TSK-13: mean 33,5 (SD 5,4) | Hospital-based interdisciplinary chronic pain center  The Auckland Regional Pain Service (TARPS) | “Participants attended twice-weekly VR appointments for 6 weeks, supervised by a physiotherapist with 4 years of experience in using VR for chronic pain. The HTC Vive immersive VR system (HTC Corporation) was used with a head-mounted display and accompanying hand sensors. The VR software programs were run via a wall-mounted desktop display that allowed the physiotherapist to view the participant’s visual field. Games that encouraged full-body movements were selected, and participants were guided to perform physically active tasks within the virtual environment and progressed through VR games at the discretion of the treating physiotherapist. 1: Fruit Ninja VR, Holodance 2: Candy smash VR 3: QuiVR, NBA 2KVR Experience 4: Lightblade VR, Bitsplan 5: Space Pirate Trainer 6: Fancy Skiing VR, Doritos VR Battle” |
| Venuturupalli et al [47] | 2019 | Virtual Reality-Based Biofeedback and Guided Meditation in Rheumatology: A Pilot Study | Mixed Methods | 20 | Age: Mean 52.65 (SD 16.1)  Rheumatoid arthiritis: n = 11  systemic lupus erythematosus: n = 4  fibromyalgia: n = 3  PROMIS scores Physical health: Mean10.94 (SD = 2.61, T = 37.4)  Mental health 12.41 (SD = 3.30, T = 43.50) Anger: 12 (SD = 3.34, T = 52.7)  Anxiety: 18.29 (SD = 4.86, T = 75.4) | Attune Health Private practice rheumatology clinic  Los Angeles, USA | “We selected two VR environments for participants based on AppliedVR’s EaseVR chronic pain platform. One module consisted of a guided meditation (GM) environment, whereas the other module consisted of a respiratory biofeedback (BFD) environment.” |
| Vugts et al [50] | 2016 | Feasibility of Applied Gaming During Interdisciplinary Rehabilitation for Patients With Complex Chronic Pain and Fatigue Complaints: A Mixed-Methods Study | Mixed Methods | 84 | Age: Mean 44.4 (SD 10.8)  Scores for depressive: Mean 42.9 (SD 11.4)  Anxious: Mean 22.2 (SD 8.2) | Facilities of Ciran  Dutch rehabilitation center | “LAKA (name of the intervention) delivers skills training with metaphorical simulation elements (encounters) and guided exercises for focused attention and open awareness. LAKA delivers skills training with metaphorical simulation elements (encounters) and guided exercises for focused attention and open awareness. These elements are interspersed with images of real-world environments, immersive mini-games, and in-game debriefings for “transferring” new insights beyond the virtual world.” |
